# A Second Look at the Crystal Structures of *Drosophila melanogaster* Acetylcholinesterase in Complex with Tacrine Derivatives Provides Insights Concerning Catalytic Intermediates and the Design of Specific Insecticides

**DOI:** 10.3390/molecules25051198

**Published:** 2020-03-06

**Authors:** Florian Nachon, Terrone L. Rosenberry, Israel Silman, Joel L. Sussman

**Affiliations:** 1 Département de Toxicologie et Risques Chimiques, Institut de Recherche Biomédicale des Armées, 91220 Brétigny-sur-Orge, France; 2 Departments of Neuroscience and Pharmacology, Mayo Clinic College of Medicine, Jacksonville, FL 32224, USA; rosenberry@mayo.edu; 3 Department of Neurobiology, Weizmann Institute of Science, 7610001 Rehovot, Israel; Israel.Silman@weizmann.ac.il; 4 Department of Structural Biology, Weizmann Institute of Science, 7610001 Rehovot, Israel; joel.sussman@weizmann.ac.il

**Keywords:** acetylcholinesterase, *Drosophila*, X-ray structures, inhibitors

## Abstract

Over recent decades, crystallographic software for data processing and structure refinement has improved dramatically, resulting in more accurate and detailed crystal structures. It is, therefore, sometimes valuable to have a second look at “old” diffraction data, especially when earlier interpretation of the electron density maps was rather difficult. Here, we present updated crystal structures of *Drosophila melanogaster* acetylcholinesterase (*Dm*AChE) originally published in [Harel et al., Prot Sci (2000) 9:1063-1072], which reveal features previously unnoticed. Thus, previously unmodeled density in the native active site can be interpreted as stable acetylation of the catalytic serine. Similarly, a strong density in the *Dm*AChE/ZA complex originally attributed to a sulfate ion is better interpreted as a small molecule that is covalently bound. This small molecule can be modeled as either a propionate or a glycinate. The complex is reminiscent of the carboxylate butyrylcholinesterase complexes observed in crystal structures of human butyrylcholinesterases from various sources, and demonstrates the remarkable ability of cholinesterases to stabilize covalent complexes with carboxylates. A very strong peak of density (10 σ) at covalent distance from the Cβ of the catalytic serine is present in the *Dm*AChE/ZAI complex. This can be undoubtedly attributed to an iodine atom, suggesting an unanticipated iodo/hydroxyl exchange between Ser238 and the inhibitor, possibly driven by the intense X-ray irradiation. Finally, the binding of tacrine-derived inhibitors, such as ZA (1DX4) or the iodinated analog, ZAI (1QON) results in the appearance of an open channel that connects the base of the active-site gorge to the solvent. This channel, which arises due to the absence of the conserved tyrosine present in vertebrate cholinesterases, could be exploited to design inhibitors specific to insect cholinesterases. The present study demonstrates that updated processing of older diffraction images, and the re-refinement of older diffraction data, can produce valuable information that could not be detected in the original analysis, and strongly supports the preservation of the diffraction images in public data banks.

## 1. Introduction

Acetylcholinesterase (AChE) terminates synaptic transmissions at cholinergic synapses by rapid hydrolysis of the neurotransmitter, acetylcholine [1]. This essential role is conserved across a large range of vertebrate and invertebrate species, including insects [2]. Anticholinesterases belonging to the organophosphate and carbamate families are one of the principal categories of insecticides utilized, and much effort has been devoted to the development of ones that are selective for insects over humans and other higher vertebrates [3]. Mutations in the AChE genes of insects (ace-1 and ace-2) resulted in the appearance of insecticide-resistant strains [4]. A solution of the crystal structure of *Drosophila melanogaster* AChE (*Dm*AChE) helped to understand and overcome the molecular basis of this resistance [5,6]. It also assisted the continued development of more selective insecticides. This remains an important issue, since it is currently estimated that exposure to AChE-targeted pesticides, whether accidental, e.g., during crop spraying, or due to suicide attempts, is responsible for 170,000 deaths yearly worldwide [7].

**Figure 1:**
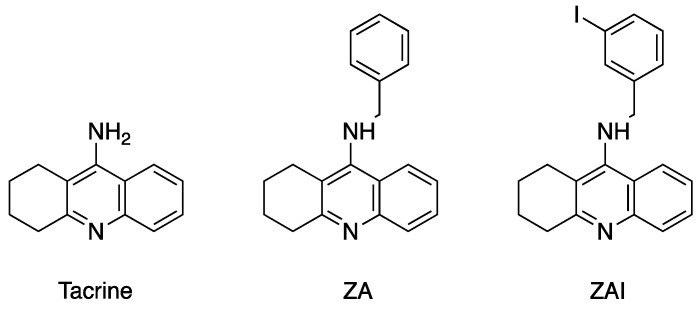
Chemical structures of acetylcholinesterase (AChE) inhibitors: tacrine, ZA and ZAI.

The first X-ray structure of an insect AChE was published almost 20 years ago, and was that of the fruit fly *Drosophila melanogaster* [5]. It revealed that the overall fold of the insect enzyme is very similar to that of vertebrate AChEs. However, the active-site gorge is much smaller, with a volume ca. 50% of that of the vertebrate AChEs, due to the presence of additional aromatic residues. The role of these aromatic residues in the binding of ligands is illustrated in the structures of complexes of *Dm*AChE with two benzyl-tacrine reversible inhibitors, ZA and ZAI (Figure 1). These structures provided the first rational basis for explaining differences in specificity and activity between *Dm*AChE and mammalian AChEs.

Recently, crystal structures of another insect AChE have been published, that of the wild type *Anopheles gambiae* AChE (*Ag*AChE) [8] and of an insecticide-resistant mutant of the same enzyme [9]. The sequence similarity of *Ag*AChE and *Dm*AChE is relatively low (41.1% identity) since they are coded by non-homologous genes, ace-1 and ace-2, respectively [9]. Their overall structures are, nevertheless, very similar (1.9 Å rmsd) [8]. The structure of *Ag*AChE notably confirmed the existence of an open channel at the base of the active-site gorge [9], as previously identified in *Dm*AChE [6]. It is suggested that this particular structural feature could be exploited to design highly specific insecticides.

Since the original publication of the *Dm*AChE structures, dramatic improvement has been made in the refinement of X-ray structures with the advent and constant improvement over time of software like XDS for diffraction image processing [10], Phenix for model refinement [11], and Coot for model building [12]. The fact that crystallographers are requested to deposit their diffraction data, alongside the structure coordinates, in the Protein Data Bank (PDB), when submitting an X-ray structure, offers the possibility for third parties to update the analysis of the data. This led to initiatives such as PDB-REDO (pdb-redo.eu), which provides a platform for optimizing structures deposited in the PDB. Going a step further, there have also been recent initiatives to deposit the diffraction images in databases (proteindiffraction.org or data.sbgrid.org), thus providing the opportunity to reprocess the raw images so as to improve both the structure factor datasets and the refined structure models. It is noteworthy that macromolecular crystallography is a rare scientific discipline in which researchers are invited to deposit their raw experimental data in the public domain, and indeed do so willingly. In the present case study, close examination of both the electron density maps and of the original models of the *Dm*AChE structures suggested that there was room for improvement. Besides, the original diffraction images collected at the Elettra synchrotron in 1998 in Trieste were still available in the electronic archive of the laboratory, providing the possibility to reprocess the images in order to improve the structure factor data. Thus, we here present optimized structures of *Dm*AChE in its native form and in complexes with ZA and ZAI.

## 2. Results and Discussion

### 2.1. Updated Image Processing and Structures Refinement

The diffraction images were still available for crystals of native *Dm*AChE and of its complex with ZAI. Two datasets collected from a single crystal of native *Dm*AChE (100 images at high resolution and 100 images at low resolution) were processed and merged using XDS. The information contained in the image header was used for the input files. The statistics of the reprocessed reflection data are reported in Table 1, and are compared to the original data. The resolution of the data improved from 2.7 to 2.45 Å, and the number of unique reflections increased by 33% without dramatic loss of completeness (98.8% to 95.3%) or I/σ ratio (5.8 to 3.3) in the highest-resolution shell. A single dataset of 70 images collected from a crystal of ZAI/*Dm*AChE was processed with XDS. The resolution improved from 2.7 to 2.5 Å, the number of unique reflections increased by 25% without any loss of completeness (98.7% to 98.4%), and with an acceptable loss in I/σ ratio in the highest-resolution shell (5.8 to 3.3). These statistics indicate a clear improvement of the overall data quality for both crystals, with more information available for the refinement of the models. No images were available for the ZA/*Dm*AChE complex; thus the original structure factors from the PDB were used in the subsequent cycles of model building and refinement.

All three structures were refined with Coot and Phenix starting from their respective coordinates deposited in the PDB. In comparison to the original refined structures, the final R-work values systematically decreased by ~15 to 25. The R-free of the ZA complex showed the most significant decrease (~10% from 0.259 to 0.233), while that of the ZAI complex decreased by 4%. Surprisingly, the R-free value of the native enzyme increased from 0.270 to 0.295. However, as calculated by Phenix, the R-free is slightly smaller than the R-work in the original structure of the native enzyme, which is abnormal and suggests a bias. It is noteworthy that the original statistics reported by Harel et al., R-work = 0.258 and R-free = 0.301, are in better agreement with the statistics of the updated native *Dm*AChE structure. The geometries of the updated structures also improved noticeably, with Root Mean Square Deviation for angles of ~1°, as compared to ~1.4°–1.8°, and dramatically improved Ramachandran, rotamer, and clashscore statistics (Table 1). However, Wilson B-factors were larger in the updated structures, which was expected, since more reflections in the highest-resolution shells were integrated into the final datasets.

All things considered, the updated structures are of much better quality statistically than the original ones, and justify the reprocessing. These improvements translate into the quality of the electron density maps and their interpretation, resulting in more accurate models, as discussed below.

### 2.2. Crystal Structure of Native Drosophila melanogaster Acetylcholinesterase

**Figure 2:**
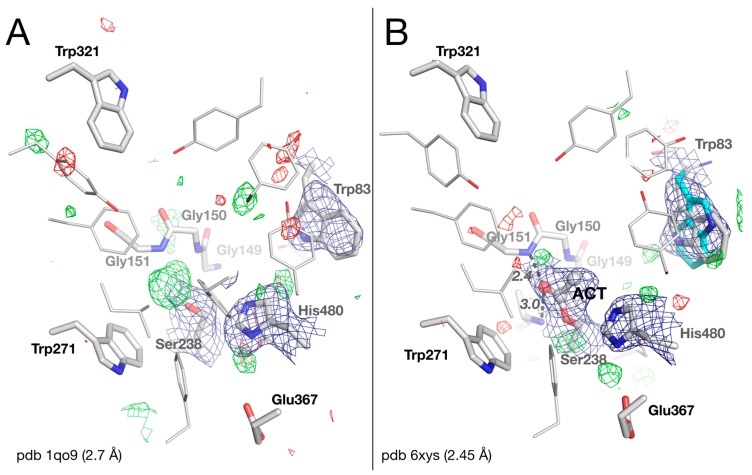
Active-site gorge in the original (**A**) and updated (**B**) structures of native *Dm*AChE. Residues of the catalytic triad (Glu367/His480/Ser238), of the oxyanion hole (Gly150/Gly151/Ala239), and key residues of the peripheral site (Trp321), acyl-binding pocket (Trp271), and choline-binding pocket (Trp83), are represented as sticks, with carbons in white, nitrogens in blue, and oxygens in red. The alternative conformation of Trp83 is depicted with carbons in cyan. The acetyl (ACT) is represented as balls and sticks. H-bonds are depicted as black dashes, with distances in Å. The meshes represent the 2 |Fo| – |Fc| map (1 σ blue) and the |Fo| – |Fc| difference map (3 σ green /−3 σ red).

An examination of the |Fo|−|Fc| difference electron density map of the original native *Dm*AChE structure (pdb 1qo9; Figure 2A) reveals a peak of positive density at 5 σ, close to Oγ of the catalytic Ser238. There is a molecule at that position that was not originally modeled. It is, in fact, not unusual to find a water molecule at this spot, bridging the catalytic serine to a glycine of the oxyanion hole, as seen, for example, in the human AChE-galantamine complex (pdb 4ey6) [14]. Yet if we model a water molecule, and refine the structure using the updated structure factors, a large unexplained positive electron density remains. The intensity and shape of this electron density peak hints that a larger molecule is present. The overlap of the electron density with Ser238Oγ also suggests that the unidentified moiety is covalently bound to the catalytic serine. Knowing that the crystals were grown in acetate buffer at pH 4.6, we chose to model the density as an acetate bound to the serine, i.e., as an acetylated serine (Figure 2B). The acetyl moiety fits nicely into the density, with the carbonyl oxygen firmly anchored in the oxyanion hole by H-bonds with the mainchain nitrogens of Gly151 and Ala239 (at distances of 2.4 and 3.0 Å, respectively). To our knowledge, this is the first case in which an acylated serine is observed in a cholinesterase, with the carbonyl carbon in a trigonal planar state. By contrast, carboxylate complexes, i.e., tetrahedral intermediates, have already been observed within the active site of human butyrylcholinesterase (BChE) [15,16]. Formation of the acetyl-serine likely results from binding of an acetate anion from the buffer to form a tetrahedral intermediate, which dehydrates due to the very acidic crystallization conditions. In this acetylated form, the methyl is not located within the acyl-binding pocket delimited by residues Trp271, Phe440, Leu328, and Phe330, but points toward His480 of the catalytic triad. His480 is not in the correct orientation for forming an H-bond with Ser238; instead, it forms a weak H-bond with Glu367 (3.4 Å). The catalytic triad is thus disrupted. Disruption of the catalytic triad also rationalizes the failure of the acyl-enzyme to hydrolyze.

Trp83 of Cys-loop 66–93, the key residue for binding the quaternary ammonium of acetylcholine, appears disordered in the maps. It was originally modeled as an unusual rotamer, with a 10° rotation around Cα-Cβ (Chi1) and 180° rotations around Cβ-Cγ (Chi2), compared to the common rotamer observed in other ChEs. In fact, during the refinement with the updated dataset, both rotamers equally fit into the 2|Fo|–|Fc| map. Thus, we chose to reflect this observation by modelling Trp83 in two alternative conformations corresponding to the two rotamers (Figure 2B). The conformational instability of Trp83 in *Dm*AChE is mostly related to the absence of a tyrosine residue that is present in vertebrates but not in insects, as discussed below and elsewhere [6,9].

### 2.3. Crystal structure of Drosophila melanogaster Acetylcholinesterase in Complex with ZA

**Figure 3:**
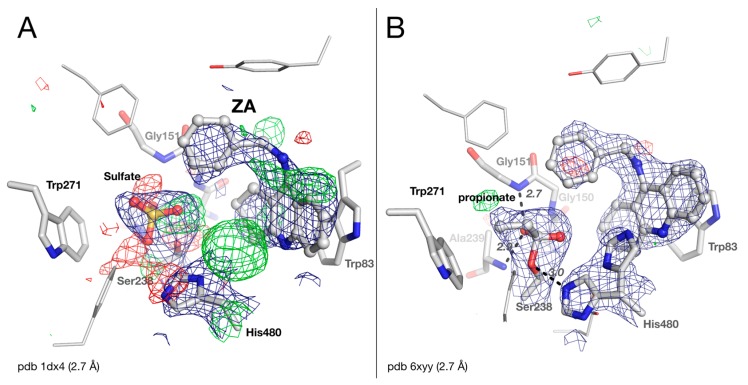
Active-site gorge of the original (**A**) and updated (**B**) structures of the complex of *Dm*AChE with ZA (benzyltacrine). Residues of the catalytic triad (His480/Ser238), the oxyanion hole (Gly150/Gly151/Ala239), and key residues of the acyl-binding pocket (Trp271) and choline-binding pocket (Trp83), are represented as sticks, with carbons in white, nitrogens in blue, and oxygens in red. The bound sulfate, propionate, and ZA are shown as ball-and-stick models. H-bonds are shown as black dashes, with distances in Å. The meshes represent the 2 |Fo| – |Fc| map (1 σ blue) and the |Fo| – |Fc| difference map (3 σ green /−3 σ red).

There were multiple modelling issues in the original structure of the benzyltacrine (ZA) complex with *Dm*AChE (Figure 3A). The geometry of the central ring of the tacrine moiety is bad: thus, there is a large positive peak in the |Fo|-|Fc| map near His480, and a sulfate ion modeled in the active site not only clashes strongly with Ser238Oγ (2.1 Å for O3, 2.4 Å for S), but is surrounded by multiple positive and negative peaks in the |Fo|-|Fc| map. We were able to solve these issues in the updated refinement (Figure 3B)**.** The density originally attributed to the sulfate ion is well interpreted as a covalently bound small molecule. In contrast to native *Dm*AChE, the molecule cannot be modeled as an acetyl ester of the active-site serine but rather as a carboxylated molecule with a 3-atom chain, such as glycine or propionate, with the carboxylic carbon at 1.6 Å distance from Ser238Oγ. We chose to model the electron density as a propionate arbitrarily, despite the fact that no propionate was present in the crystallization buffer. This situation, in which a carboxylate of unknown origin is modelled in the active site of a cholinesterase, is similar to that observed earlier for carboxylate-human butyrylcholinesterase complexes [15,16]. This carboxylate-*Dm*AChE complex is strikingly similar to carboxylate-human butyrylcholinesterase complexes except for the carboxylic carbon-serine distance that is noticeably longer in the latter (2.1 Å). One carboxylate oxygen is anchored in the oxyanion hole by H-bonds with the mainchain nitrogens of Gly151 and Ala239 (at distances of 2.7 and 2.9 Å, respectively). This observation again demonstrates the remarkable ability of ChEs to form stable complexes with carboxylates, as supported by quantum mechanical calculations [17] and secondary kinetic isotope effect experiments [18].

The strong density peak at 8 σ, near His480, is well interpreted as an alternate conformation of this residue. In conformation A, the imidazole ring is in close contact with ZA (3.2 Å between His380Nε2 and N10 of ZA), and the catalytic triad is disrupted. In conformation B, the imidazole ring is oriented in the classical triad conformation with His380Nε2 at 3.0 Å from Ser238Oγ, and His380Nε2 at 2.4 Å from Glu367Oε1. The catalytic histidine of *Dm*AChE is mobile, as observed for both *Tc* and mouse AChE [19,20], but not for the histidines of human AChE or BChE [21,22].

### 2.4. Crystal Structure of Drosophila melanogaster Acetylcholinesterase in Complex with ZAI

**Figure 4:**
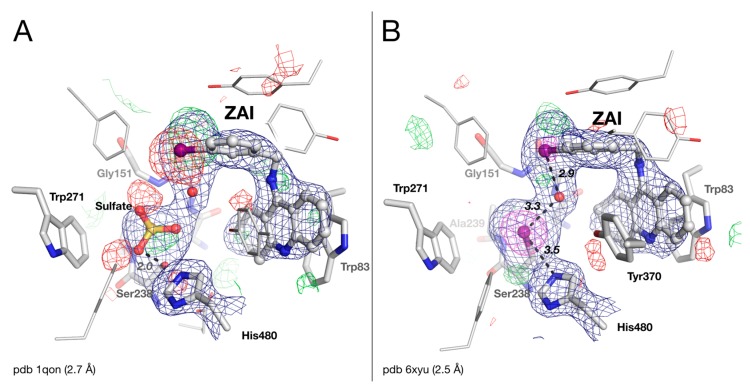
Active-site gorge of the original (**A**) and updated (**B**) structures of *Dm*AChE in complex with ZAI (iodobenzyltacrine). Residues of the catalytic triad (His480/Ser238), of the oxyanion hole (Gly150/Gly151/Ala239), and key residues of the acyl-binding pocket (Trp271) and choline-binding pocket (Trp83), are represented as sticks, with carbons in white, nitrogens in blue, and oxygens in red. The bound sulfate, iodine and ZAI are represented as ball-and-stick models. H-bonds are shown as black dashes, with distances in Å. The meshes represent the 2|Fo| – |Fc| map (1 σ blue / 5 σ magenta) and the |Fo| − |Fc| difference map (3 σ green /−3 σ red).

The crystal structure of the *Dm*AChE/ZAI complex was refined with the updated dataset. Some positive and negative peaks in the |Fo|-|Fc| map seen in the original structure near the iodine atom of ZAI (Figure 4A) are easily corrected by shifting the iodobenzyl ring by ~1 Å (Figure 4B). As noted by Harel et al. [6], the occupancy of the iodine atom of the ligand has to be decreased to 0.5, so as to minimize peaks in the |Fo|-|Fc| map, supporting the loss of 50% of the iodine, likely due to radiation damage from the intense synchrotron X-rays [23,24].

As in the *Dm*AChE/ZA complex, the sulfate ion originally modeled in the active site of the *Dm*AChE/ZAI complex clashes strongly with Ser238Oγ (2.0 Å for O3, 2.3 Å for S), with some positive and negative peaks in the |Fo|-|Fc| map (Figure 4A). Removing the sulfate ion, and refining the structure with the updated dataset, reveals a 10 σ density peak in the |Fo|-|Fc| map, by far the strongest in the map, very close to Ser238Cβ (2.1 Å). The density has a ball shape, unlike that observed for the propionate in *Dm*AChE/ZA structure. It can be plausibly modeled as an iodine atom with full occupancy, lost by ZAI, and covalently bound to Ser238Cβ, i.e., Ser238 is converted to an iodoalanine residue (Figure 4B). The most likely explanation for the formation of this iodoalanine is that a iodo/hydroxyl exchange occurs between Ser238 and ZAI during data collection, produced by the intense X-ray irradiation. Indeed, X-ray synchrotron radiation has been reported to cleave bonds between carbon and heavy halogen atoms, such as bromine or iodine [25], forming highly reactive radical species [23,24]. The Ser238Cβ-I distance was refined to 2.1 Å, which is standard for a C-I bond, and the iodine atom is at halogen bond distance both from His480Nε2 (3.5 Å) and from a water molecule (3.3 Å) [26]. This water molecule is also at halogen bond distance from the iodine of ZAI (2.9 Å).

It is noteworthy that His480 does not adopt an alternative conformation like that seen in the *Dm*AChE/ZA complex. This alternative conformation is prevented by the conformational orientation of Tyr370, in aromatic stacking with the 4-aminoquinoline moiety of ZAI (3.8 Å inter-ring distance). This conformation of Tyr370 is similar to the conformation of the equivalent tyrosine in human and mouse AChEs in complex with huprines or tacrine [27], and could be favored by a weak H-bond between Tyr370OH, and the water molecule close to the iodine (3.4 Å).

### 2.5. Specific Channel in the Choline-Binding Pocket of Insect Cholinesterase

**Figure 5:**
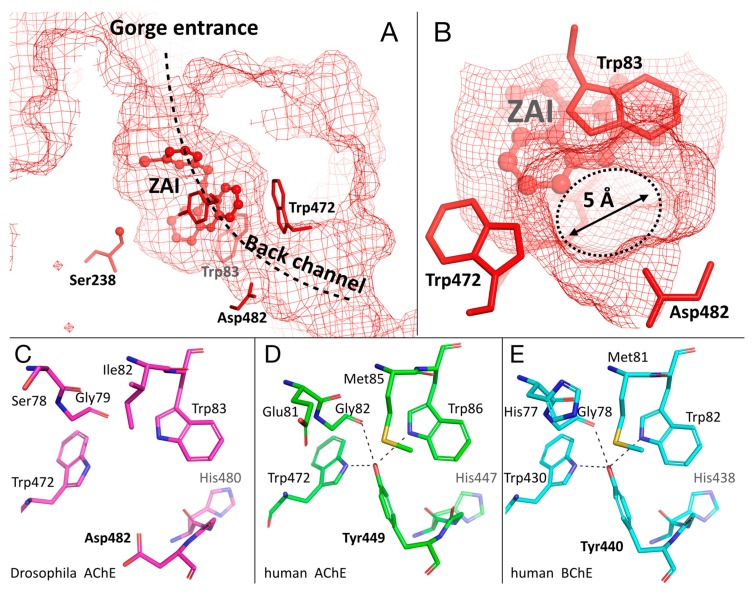
Sideview (**A**) and rearview (**B**) of the channel going through the active-site gorge in the *Dm*AChE/ZAI complex. The residues at the gate of the channel opening, near the bottom of the gorge, are represented as sticks (Trp83/Trp472/Asp82). The solvent-accessible surface is represented by a mesh. Comparison of the channel opening region of *Dm*AChE (**C**), and the homologous regions in human AChE (**D**) and human BChE (**E**). Residues of interest are represented as sticks, and important H-bonds as black dashes.

The binding of the benzyltacrine inhibitors, ZA and ZAI, stabilizes Trp83 in the consensus conformation seen in the ChEs (Figure 3B and Figure 4B). This conformation results in the opening of a 5-Å diameter pore in the gorge, delimited by Trp83, Trp472, and Asp482 (Figure 5B). The pore connects the bottom of the gorge to the bulk solvent, and forms the extremity of a channel extending from the gorge entrance through to the active site (Figure 5A). This channel was not noticed by Harel et al. [6] but was later shown to be devoid of relevance in regard to substrate trafficking [6]. A comparison of the back-channel region of *Dm*AChE and the homologous region of human AChE/BChE shows that a critical tyrosine is replaced by an aspartate in *DmAChE* (Figure 5C–E; Asp482). The absence of the tyrosine is at the origin of the pore, and causes a lack of stabilization of Trp83. In human AChE, the hydroxyl of Tyr449 is engaged in three H-bonds with Trp86Nε, Trp449Nε, and Gly82O (Figure 5D). In addition, Trp86 is stabilized by π-sulfur interaction with Met85. Trp82 of human BChE has a similar system of stabilization with additional π-sulfur interactions, with Met81 being sandwiched between His77 and Trp82 (Figure 5E).

The opening of a similar pore in human AChE/human BChE would require a conformational change of Tyr449 (440) which appears to be energetically unfavored by the system of stabilization described above. However, a rotation of the tyrosine, accompanied by pore opening, was observed upon binding of an antibody fragment to *Bungarus fasciatus* AChE, or of a small molecule to *Torpedo californica* AChE [28,29].

Recently, a similar channel opening was described in the crystal structure of another insect AChE, *Anopheles gambiae* AChE [9]. As in *Dm*AChE, the lack of a tyrosine, also replaced by an aspartate residue (Asp602), was found at the origin of the pore (not shown). It is noteworthy that, unlike in the case of *Dm*AChE, the key tryptophan in the choline-binding pocket of *Ag*AChE (Trp245) is stabilized by a π-sulfur interaction with a methionine (Met244). Consequently, no conformational heterogeneity was reported for Trp245.

**Figure 6:**
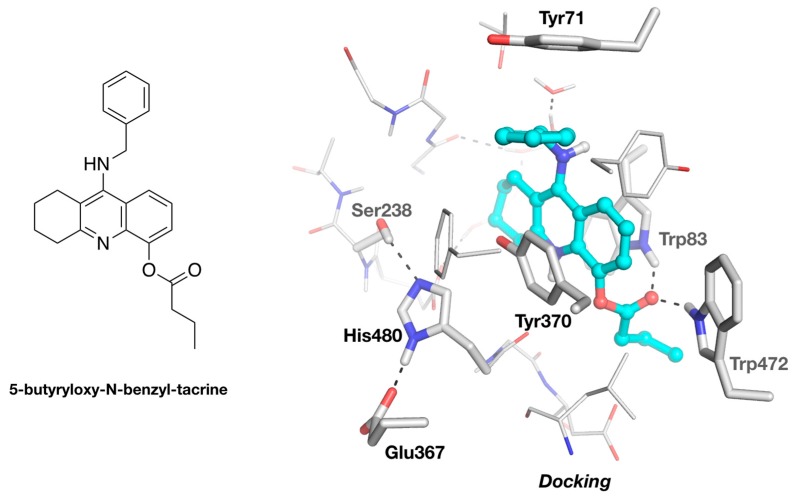
Docking of 5-butyryloxy-*N*-benzyl-tacrine into the active-site gorge of *Dm*AChE. The docking was performed with Autodock Vina [30], using the structure of *Dm*AChE/ZAI as the template (see Methods). The ligand is represented as a ball-and-stick model, with carbons in cyan. The catalytic triad and residues interacting with the ligand are represented as sticks. H-bonds are represented by black dashes.

Cheung and colleagues also noticed that the existence of the pore is specific to the mosquito enzyme *Ag*AChE vs mammalian AChE [9]. The results reported herein suggest that this specificity extends to other insects. It is tempting to exploit this feature to design a specific inhibitor of insect AChE. We tested this hypothesis in silico by designing a molecule based on ZA, by adding a long substituent on the aromatic ring close to the pore. A butyryloxy substituent appeared to be a good candidate, because it is long enough to create steric hindrance in the active site of an AChE devoid of a pore, and also it provides an additional H-bond acceptor/donor to further strengthen the enzyme/ligand interaction. Thus, we performed the docking of 5-butyryloxy-*N*-benzyl-tacrine as a specific insecticide candidate (Figure 6).

The template used to perform the docking simulation was the structure of *Dm*AChE/ZAI with the restored active-site serine, because the conformation of Tyr370 in this structure provides additional aromatic interaction with the 4-aminoquinoline moiety. The affinity given by the scoring function of Autodock Vina is in the same range as the affinity of the original inhibitor, ZA (9–10 kcal/mol), thus showing that the addition of the substituent is not detrimental to binding. As expected, the binding conformation of 5-butyryloxy-*N*-benzyl-tacrine is very similar to that of ZAI, with the benzyl aromatic ring stacked against Tyr370, and the tacrine ring in aromatic stacking with Tyr370 and Trp83 (Figure 6). As we envisioned, the butyryloxy substituent protrudes through the pore. Interestingly, the carbonyl makes two H-bonds with the indole rings of Trp83 and Trp472, thus partially replacing the contribution of the absent tyrosine. This simple example illustrates the potential of the presence of the specific back door channel to permit the design of new specific inhibitors of some insect AChEs.

### 2.6. Impact of Preserving Original Date for Potential Reinterpretation

The present study shows the great value of preserving the original raw diffraction images. In this case, it made it possible to utilize recent, higher quality software, both to process the images and to obtain improved structure factors. Consequently, through using current refinement and molecular graphics tools, it was possible to interpret the data more meaningfully. Thus, this study serves as a paradigm for the successful utilization of the original data, thus showing that it is indeed crucial to preserve them.

## 3. Materials and Methods

### 3.1. X-ray Data Processing and Structure Refinement

The diffraction images from crystals of native *Dm*AChE, and of its complex with ZAI, were both collected at Elettra, as described by Harel et al. [5]. The images were reprocessed with XDS [10], intensities of integrated reflections were scaled using XSCALE, and structure factors were calculated using XDSCONV. As diffraction images from crystals of *Dm*AChE in complex with ZA were no longer available, the structure factors were directly downloaded from the PDB. The structure determination and refinement was performed using the Phenix suite of software [11]. The structures of the native and ZAI structures were solved with phaser-MR [31] included in Phenix, using PDB entry 1QON as the starting model. For the ZA-*Dm*AChE complex, the starting model was the original PDB entry 1DX4. The starting models were refined by iterative cycles of model building with Coot [12], then restrained and subjected to TLS refinement with Phenix. The ligands and their descriptions were built using phenix.elbow which is included in Phenix. Data collection and refinement statistics, as calculated using Phenix, are shown in Table 1. The protein structures were illustrated using the program PyMOL (Schrödinger).

### 3.2. Molecular Docking of 5-Butyryloxy-9-Benzyltacrine in DmAChE

The molecular docking was performed using AutoDock Vina [30]. The system was prepared in PyMOL (Schrödinger), using the plug-in developed by Daniel Seeliger (https://github.com/ADplugin/ADplugin). *Dm*AChE was constructed from the structure of the *Dm*AChE/ZAI complex, mutating the iodoalanine back to a serine, and retaining the structurally relevant water molecules lining the gorge. A 24 × 24 × 24 Å docking box was employed, centered near the hydroxyl of Tyr370. ZA and 5-butyryloxy-9-benzyltacrine were built and optimized from their SMILE string, using phenix.elbow [11]. The default parameter set of AutoDock Vina was used to generate 9 docking poses. The pose with the best energy score was selected as the most representative.

## Figures and Tables

**Table 1:** Data collection and refinement statistics. Calculated using Phenix [13]. R-work = Σ |fo| − |fc| / Σ |fo|, fo and fc are observed and calculated structure factors. Statistics for the highest-resolution shell are shown in parentheses.

Ligand	Native		ZAI		ZA	
pdb code	1qo9	6xys	1qon	6xyu	1dx4	6xyy
**Software**						
Integration	DENZO	XDS	DENZO	XDS	DENZO	
Scaling	SCALEPACK	XSCALE	SCALEPACK	XSCALE	SCALEPACK	
Refinement	CNS	Phenix	CNS	Phenix	CNS	Phenix
Model building	O	Coot	O	Coot	O	Coot
**Data Collection**						
X-ray source—beamline	Elettra, Trieste, XRD		Elettra, Trieste, XRD		NSLS, BNL, X12C	
wavelength (Å)	1.0		1.0		0.98	
Resolution range (Å)	29.83–2.7	30.94– 2.45	26.54–2.707	40.82–2.51	29.78–2.7	
(highest-resolution shell)	(2.796–2.7)	(2.543–2.455)	(2.804–2.707)	(2.6–2.51)	(2.796–2.7)	
space group, mol/AU	P4_3_2_1_2, 1	P4_3_2_1_2, 1	P4_3_2_1_2, 1	P4_3_2_1_2, 1	P4_3_2_1_2, 1	
unit cell parameters (Å)	94.3 94.3 159.0 90 90 90	94.6 94.6 159.0 90 90 90	94.9 94.9 160.0 90 90 90	94.9 94.9 160.0 90 90 90	95.8 95.8 162.0 90 90 90	
Total reflections	na	161402 (9870)	na	70404 (6919)	na	
Unique reflections	20199 (1956)	26803 (2542)	19963 (1998)	24909 (2458)	20596 (1809)	
Multiplicity	na	6.0 (3.9)	na	2.8 (2.8)	na	
Completeness (%)	98.95 (98.79)	98.54 (95.29)	96.58 (98.71)	96.64 (98.40)	95.98 (86.67)	
Mean I/σ (I)	33.5 (5.8)	20.7 (3.3)	15.5 (2.4)	10.97 (1.68)	24.3 (3.2)	
Wilson B-factor	45.34	51.47	49.70	52.05	50.19	
R-merge	na	0.0487 (0.335)	na	0.0635 (0.519)	na	
R-meas	0.05 (0.161)	0.0524 (0.387)	0.060 (0.254)	0.0766 (0.638)	0.038 (0.274)	
R-pim	na	0.0184 (0.1848)	na	0.0412 (0.358)	na	
CC1/2	na	0.999 (0.939)	na	0.997 (0.763)	na	
CC*	na	1 (0.984)	na	0.999 (0.93)	na	
**Refinement Statistics**						
Reflections used	20196 (1956)	26739 (2530)	19930 (1996)	24876 (2457)	20578 (1807)	20577 (1807)
Reflections for R-free	1990 (176)	1338 (126)	1704 (152)	870 (86)	2030 (193)	2030 (193)
R-work	0.2728 (0.4117)	0.2297 (0.3959)	0.2325 (0.3431)	0.1857 (0.3252)	0.2241 (0.3180)	0.1711 (0.2461)
R-free	0.2701 (0.4481)	0.2950 (0.5239)	0.2604 (0.3615)	0.2522 (0.4266)	0.2595 (0.3321)	0.2335 (0.3270)
Number of non-H atoms	4479	4398	4486	4447	4413	4483
macromolecule	4273	4239	4258	4230	4231	4242
ligands	75	65	89	63	91	97
solvent	131	94	139	154	97	144
Protein residues	540	542	686	542	537	537
RMSD (bonds; Å)	0.010	0.008	0.011	0.008	0.008	0.008
RMSD (angles; deg)	1.81	1.05	1.54	0.96	1.41	0.90
Ramachandran favored (%)	77.99	90.96	88.81	94.93	88.93	95.12
Ramachandran allowed (%)	16.04	8.10	9.33	4.50	9.38	4.50
Ramachandran outliers (%)	5.97	0.94	1.87	0.56	1.69	0.38
Rotamer outliers (%)	10.34	0.22	7.16	0.00	6.28	0.22
Clashscore	64.63	18.47	35.07	10.40	28.62	7.45
Average B-factor (Å^2^)	58.32	79.57	54.32	66.71	50.47	56.87
macromolecules (Å^2^)	58.43	79.61	54.02	66.59	50.15	56.43
ligands (Å^2^)	85.59	102.26	84.26	85.23	75.02	85.34
solvent (Å^2^)	39.15	61.86	44.27	62.47	40.67	50.57
Number of TLS groups	-	5	-	3	-	4
